# “Quitlink”—A Randomized Controlled Trial of Peer Worker Facilitated Quitline Support for Smokers Receiving Mental Health Services: Study Protocol

**DOI:** 10.3389/fpsyt.2019.00124

**Published:** 2019-03-19

**Authors:** Amanda L. Baker, Ron Borland, Billie Bonevski, Catherine Segan, Alyna Turner, Lisa Brophy, Kristen McCarter, Peter J. Kelly, Jill M. Williams, Donita Baird, John Attia, Rohan Sweeney, Sarah L. White, Sacha Filia, David Castle

**Affiliations:** ^1^Faculty of Health and Medicine, University of Newcastle, Newcastle, NSW, Australia; ^2^Cancer Council Victoria, Melbourne, VIC, Australia; ^3^Melbourne School of Population and Global Health, The University of Melbourne, Melbourne, VIC, Australia; ^4^IMPACT Strategic Research Centre, School of Medicine, Barwon Health, Deakin University, Geelong, VIC, Australia; ^5^Department of Psychiatry, University of Melbourne, Melbourne, VIC, Australia; ^6^Mind Australia Limited, Melbourne, VIC, Australia; ^7^School of Allied Health, Human Services and Sport, La Trobe University Melbourne, Melbourne, VIC, Australia; ^8^Illawarra Health and Medical Research Institute and the School of Psychology, University of Wollongong, Wollongong, NSW, Australia; ^9^Division of Addiction Psychiatry, Rutgers Robert Wood Johnson Medical School, New Brunswick, NJ, United States; ^10^Centre for Health Economics, Monash University, Melbourne, VIC, Australia; ^11^Department of Psychiatry, St Vincent's Hospital Melbourne, Fitzroy, VIC, Australia

**Keywords:** smoking, smoking cessation, quitline, peer worker, mental illness, severe mental illness, psychosis, depression

## Abstract

**Introduction:** Although smokers with severe mental illnesses (SSMI) make quit attempts at comparable levels to other smokers, fewer are successful in achieving smoking cessation. Specialized smoking cessation treatments targeting their needs can be effective but have not been widely disseminated. Telephone delivered interventions, including by quitlines, show promise. However, few SSMI contact quitlines and few are referred to them by health professionals. Mental health peer workers can potentially play an important role in supporting smoking cessation. This study will apply a pragmatic model using peer workers to engage SSMI with a customized quitline service, forming the “Quitlink” intervention.

**Methods:** A multi-center prospective, cluster-randomized, open, blinded endpoint (PROBE) trial. Over 3 years, 382 smokers will be recruited from mental health services in Victoria, Australia. Following completion of baseline assessment, a brief intervention will be delivered by a peer worker. Participants will then be randomly allocated either to no further intervention, or to be referred and contacted by the Victorian Quitline and offered a targeted 8-week cognitive behavioral intervention along with nicotine replacement therapy (NRT). Follow-up measures will be administered at 2-, 5-, and 8-months post-baseline. The primary outcome is 6 months continuous abstinence from end of treatment with biochemical verification. Secondary outcomes include 7-day point prevalence abstinence from smoking, increased quit attempts, and reductions in cigarettes per day, cravings and withdrawal, mental health symptoms, and other substance use, and improvements in quality of life. We will use a generalized linear mixed model (linear regression for continuous outcomes and logistic regression for dichotomous outcomes) to handle clustering and the repeated measures at baseline, 2-, 5-, and 8-months; individuals will be modeled as random effects, cluster as a random effect, and group assignment as a fixed effect.

**Discussion:** This is the first rigorously designed RCT to evaluate a specialized quitline intervention accompanied by NRT among SSMI. The study will apply a pragmatic model to link SSMI to the Quitline, using peer workers, with the potential for wide dissemination.

**Clinical Trial Registration:** **Trial Registry:** The trial is registered with ANZCTR (www.anzctr.org.au): ACTRN12619000244101 prior to the accrual of the first participant and updated regularly as per registry guidelines.

**Trial Sponsor:** University of Newcastle, NSW, Australia.

## Introduction

Smokers living with severe mental illness (SSMI) die around two decades earlier than the general population, due largely to smoking-related diseases (1). Survey data from the United States (2, 3), United Kingdom (4), and Australia (5) consistently highlight that smoking is not declining at the same rate among SSMI as among the general population. In Australia, smoking rates in 2010 for people with psychotic illness were 67 vs. 65% in 1997/98 (5). Rates of smoking increase with severity of the mental illness. The Australian National Drug Strategy Household Survey (6) showed that in 2016, among those diagnosed with a mental illness in the previous 12 months, daily smoking rates were highest among people with a psychotic disorder (schizophrenia 49.3%, bipolar 37.3%, other psychotic disorder 32.2%), followed by anxiety disorders (28.5%), depression (27.3%), and eating disorders (24%). In comparison, 12% of people from the general population in Australia smoke daily (7). Economic costs associated with smoking in people with mental illness are significant and include costs of treatment of tobacco-related diseases, work-related absenteeism, and premature mortality. In the UK, in the 2009/2010 financial year, the estimated economic cost of smoking in people with mental disorders was at £2.3 billion (8).

There is a vicious cycle between smoking and poorer mental and physical health. In addition to increased mortality, SSMI have more psychiatric symptoms, increased hospitalizations, and the requirement for higher doses of some psychiatric medications (9). This is because components of tobacco smoke accelerate their metabolism (10). Smoking cessation benefits mental health as well as physical health. A recent meta-analysis of primarily non-controlled trials found that quitting smoking is associated with significantly improved quality of life and reduced depression, mixed anxiety/depression, and improved positive affect. Effect sizes for these differences were as large in people with SMI (0.40, −0.39, −0.21, 0.68, respectively) as in the general population (0.15, −0.30, −0.32, 0.16). The effect sizes were equal to or larger than those of people receiving anti-depressant treatment for mild to severe depression (range: −0.17 to −0.11) and generalized anxiety disorder (range: −0.23 to −0.50) (11). In addition, the recent Evaluating Adverse Events in a Global Smoking Cessation Study (EAGLES) randomized controlled trial (RCT) found no significant difference in the occurrence of adverse events between the smoking cessation medications, varenicline, bupropion, nicotine replacement therapy (NRT), or placebo (12) among people with or without psychiatric disorders. Further, sustained reductions in smoking have important financial benefits and may increase the chance of future cessation (13).

SSMI are about as likely to want to quit as those in the general population (14), and some are able to quit (9). However, they often require additional assistance, and overall have lower rates of success with cessation. This can potentially be reduced or eliminated by the delivery of additional assistance targeted to their specific needs. SSMI are not uniformly being provided with the additional smoking cessation assistance they need. The problem is often overlooked by health care providers, being seen as either too hard or a low priority (15, 16), while SSMI report a lack of encouragement to quit (16). Mental health staff have reported that they lack knowledge about tobacco dependence and smoking's relationship with mental illness (17). In addition, existing evidence-based interventions for this population are rare, mainly face-to-face and intensive, so without substantial additional resources, they cannot feasibly be taken up by mental health services.

Telephone smoking cessation support tailored for SSMI may improve access and enhance cessation (18). Quitline smoking cessation counseling is effective in the general population (19). A 2013 Cochrane review found that proactive telephone counseling (where the counselor initiates one or more calls to provide support) is more effective, with better outcomes than a single-session reactive support call or brief interventions (19). Quitlines offer enormous potential for SSMI. As an existing service that can be accessed from the community, quitlines can offer an intervention for SSMI that does not require a significant increase in resources.

We are aware of three RCTs that have evaluated the effectiveness of quitline interventions among SSMI. In an under-powered pilot randomized trial (*N* = 123) among a community mental health sample, Morris et al. (20) reported that five quitline sessions plus NRT were equivalent to a much more intensive intervention consisting of five quitline sessions plus NRT and also 10 group face-to-face sessions. A breath carbon monoxide (CO) verified point prevalence abstinence rate of 10% overall was achieved at 6 months. In a second RCT, Van der Meer and colleagues compared a standard quitline service to standard quitline service plus a mood management component for callers with past major depression (*N* = 485) (21). Participants in both conditions were advised to use pharmacological aids for cessation if they smoked more than 10 cigarettes per day. Cessation rates were higher for the treatment group who received the additional mood module (31 vs. 22% at 6 months and 24 vs. 14% at 12 months), but biochemical verification was available only for a small sub-sample. In the third RCT, Rogers et al. (22) compared standard quitline counseling with specialized quitline counseling developed for smokers attending Veterans Health Administration mental health facilities (*N* = 577), referred via electronic medical record consult. Participants in the specialized counseling condition were significantly more likely to report 30-day abstinence compared to the standard quitline (26 vs. 18%) at 6 months. Together, these three RCTs suggest that telephone interventions accompanied by smoking cessation medication among SSMI are likely to be effective and that tailoring the intervention specifically for mental health symptomatology is likely to enhance results. We are aware of no adequately powered RCT evaluating a tailored quitline intervention (addressing mood and other symptomatology) accompanied by NRT, for people drawn from mental health services, with biochemical verification of self-reported abstinence. The present study aims to address this gap.

In Australia, the Victorian Quitline has been building counselor skills in order to support SSMI better. Segan et al. found that a specialized quitline intervention for smoking cessation among people with depression was workable, valued by smokers, and increased the probability of quit attempts (23). They then integrated key elements of our mainly telephone delivered smoking cessation intervention, demonstrated to be as effective as a more intensive face-to-face delivered intervention, among people with psychotic disorders (24, 25). The revised tailored Victorian Quitline intervention includes structured monitoring of mental health symptoms, nicotine withdrawal symptoms, and medication side-effects (26). These procedures help to distinguish temporary withdrawal symptoms from psychiatric symptoms and facilitate targeted treatment. Feedback indicated that the structured monitoring, combined with Quitline's established focus on the relationship between smoking and mood control, had a high level of acceptance by both Quitline counselors and clients and led to better integration of quitting with management of ongoing mental health conditions (26). The resulting tailored smoking cessation intervention, when coupled with peer referral, is called Quitlink, and if shown to be effective, is likely to be widely adopted.

Despite promising work in providing a more appropriate, supportive, and engaging service, quitlines remain underutilized in part because health professionals lack awareness of their free callback service and its expertise in helping SSMI (27). Mental health peer workers can potentially play an important role in supporting smoking cessation. Peer workers have been a mental health consumer or carer and this lived experience along with their training allows them to be highly credible sources of support for SSMI (28). Peer support, which is one element of peer work, is based on the belief that people who have faced, endured, and overcome adversity can offer useful support, encouragement, hope, and mentorship to others facing similar situations (29). As part of the recovery-oriented practice framework (encompassing principles of self-determination and personalized care (30), people with their own lived experience of mental ill-health and recovery (i.e., peer workers) are viewed as highly valuable members of the mental health workforce (31). The peer workforce is the most rapidly growing workforce in the Australian mental health sector, with existing research examining peer workers finding they are effective (32). Peer workers are strong role models, and are particularly successful in developing hope, promoting self-esteem, and empowering consumers (32). These unique skills are likely to be extremely valuable in helping to promote engagement of SSMI with quitline services (29).

Communication between quitline and the mental health service will be a key component of this link. The mental health service provider (utilizing peer workers) will identify smokers who may benefit from intervention and peer workers will make the referral to the Victorian Quitline for proactive telephone counseling (accompanied by free NRT). With the permission of the participant, quitline counselors will keep peer workers and other mental health professionals updated as to quitline participation. This project will examine program and cost-effectiveness of “Quitlink” for people with SMI compared with standard smoking care.

An important component of the present research is a nested qualitative study exploring experiences of peer workers, mental health staff, and participants (from both intervention and control arms), including those participants who do and do not stop smoking and/or engage with the quitline service. We will also explore in detail the barriers to cessation people face and the impact of smoking culture, support people (carers/family/partners) and other factors on intervention uptake, ongoing participation, and outcome. Future dissemination, ongoing development of resources, and improvement of our intervention will be informed by an enhanced understanding of the mechanisms by which the intervention is effective as well as refining who is likely to be successful, and why.

## Aims

The primary aim of this research is to test the effectiveness of the Quitlink intervention for smoking cessation among SSMI. It is hypothesized that the intervention will be associated with higher rates of continued abstinence from smoking following the end of the treatment period, relative to the control condition. Secondary aims are to examine: (i) the cost-effectiveness of Quitlink compared to the control condition; (ii) barriers and enablers to making and sustaining quit attempts using qualitative methods so as to better understand why cessation rates among SSMI remain low; and (iii) the effect of Quitlink on 7-day point prevalence abstinence at 8-months, and effects on reported cigarette consumption, rates of quit attempts, nicotine withdrawal, expenditure on cigarettes, smoking cessation motivation and self-efficacy, mental health, quality of life and alcohol, and cannabis use. We will also assess process measures such as extent of use of advice and use of quit smoking medications (regardless of condition). The cost-effectiveness protocol is described elsewhere by Sweeney et al. (in submission) and the nested qualitative study and outcome measures are described below.

## Methods

### Design

**Figure 1:**
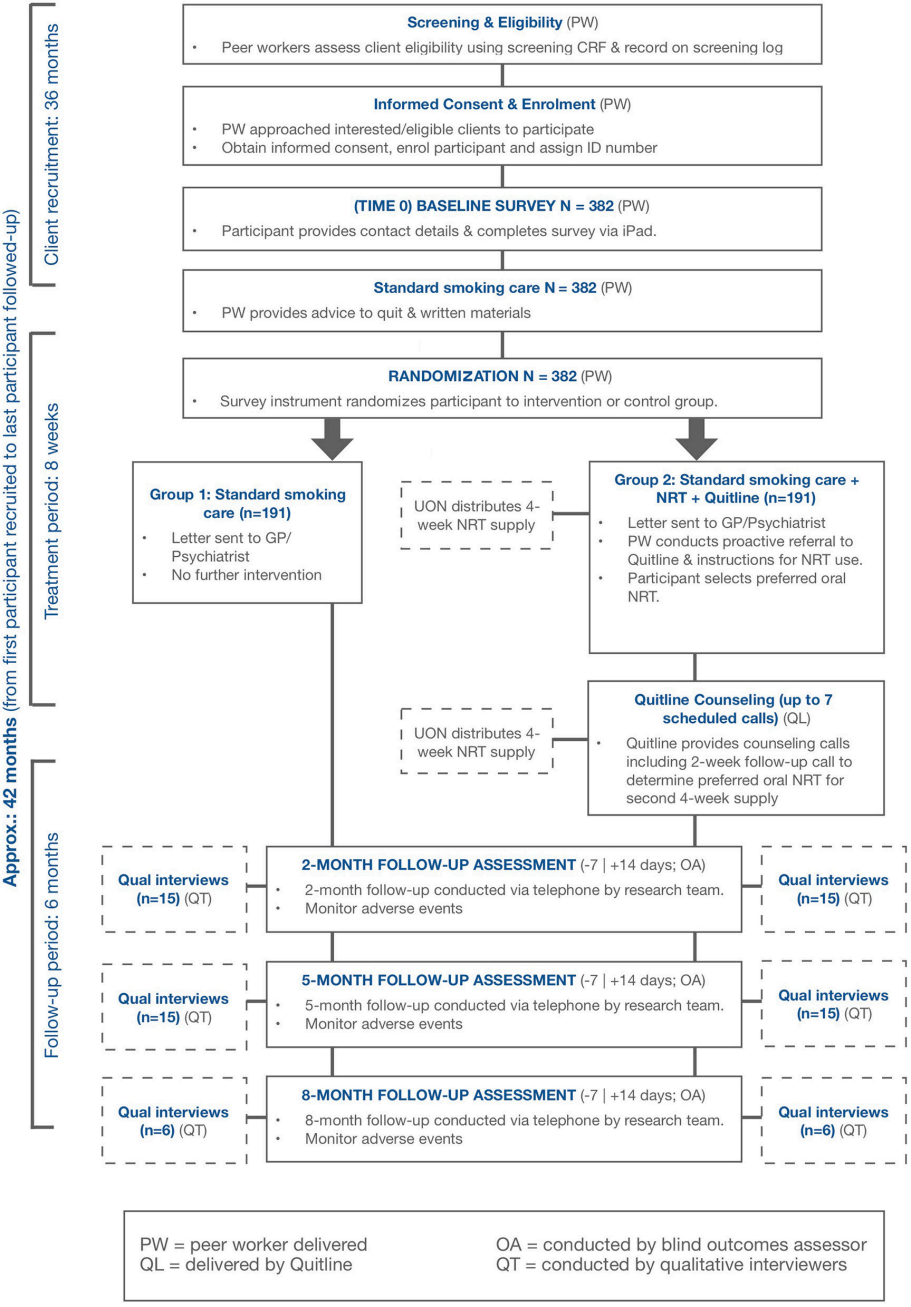
Quitlink study design.

A multi-center prospective, cluster-randomized, open, blinded endpoint (PROBE) design will be employed to compare standard smoking care alone against Quitlink. See Figure 1 for an overview.

#### Rationale for Study Design

Due to the nature of the intervention under investigation (i.e., linking smokers to existing smoking cessation care options readily available in the community, quitline, and NRT) there is a high risk of contamination among residential services where participants are living under the same roof and hence may compare treatment received. Therefore, we will use a partial clustering design where cluster randomization will be used in situations where risk of contamination is particularly high (e.g., in residential services). Individual randomization will be used in settings where participants are approached individually (e.g., clinics). Conceptually, this can be considered as a cluster RCT where some clusters only contribute a single person (see statistical methods section). This is sometimes called a split-plot design (33).

### Setting

Participants will be recruited across participating community mental health services in Victoria, Australia, including St Vincent's Mental Health, and non-government organizations such as Mind Australia Limited. Residential and non-residential community services will be included in the study. These services are widely distributed across the state and we will recruit from an as yet unknown subset of sites. The majority of people accessing these services will have been diagnosed with severe mental illnesses such as schizophrenia, schizoaffective disorder, bipolar disorder, delusional disorder, and depressive disorders.

### Eligibility Criteria

Participant inclusion criteria are: aged at least 18 years; residing in Victoria; smoking at least 10 cigarettes per day; and accessing treatment or support from participating mental health agencies. Exclusion criteria are: current engagement in Victorian Quitline's callback service; no ready access to a telephone; inability to complete informed consent and/or the screening survey; acute suicidality; myocardial infarction or unstable arrhythmia or angina within the previous 2 weeks (NRT contraindications); and pregnancy (as smokers who are pregnant already receive a different extended Quitline callback service).

Around three-quarters of individuals accessing mental health services own mobile phones (34). Landlines will be used where people do not own mobile phones, as in our previous studies (25, 35).

Quitline counselors: Experienced quitline counselors who have been provided with specialist training on counseling people with mental health issues, and who have demonstrated competence in this work have been allocated to the study (one dedicated counselor per caller for all calls, or to co-ordinate with a substitute where they may be unavailable for some calls).

### Standard Smoking Care

An active control condition, involving brief advice on the importance of quitting and provision of printed information on where to access assistance, is being utilized in this study as it reflects what is expected of mental health services as part of routine care (even though it is not routinely delivered).

The brief intervention provided to all participants includes advice to quit, encouragement to use NRT, and a Quit Victoria pack of written materials to motivate a quit attempt (e.g., costs of smoking and benefits of quitting) and resources to support self-management (e.g., Quitline phone number, “4Ds” strategy: ‘Delay, Deep-breathe, Drink water, Do something else’ to manage cravings; using NRT products).

With consent, a letter will be sent by the research team to nominated health professionals general practitioner (GP)/psychiatrist with information about their client's trial participation and a link to Australia's smoking cessation guidelines for health professionals, which includes a list of medications affected by smoking. No further intervention will be provided as part of the project for those in the control condition.

### Quitlink Intervention

The Quitlink intervention consists of all of the above plus:

- Referral to Quitline: immediately following the brief intervention, the peer worker will make a proactive referral to Quitline.
- Manual guided Quitline counseling: Quitline will then call the participant to offer the Quitlink service. This service includes up to seven scheduled calls with additional calls allowed to deal with relapse crises within an 8-week period. It includes structured monitoring of mental health symptoms, nicotine withdrawal symptoms, and medication side-effects; and a focus on psychoeducation including the relationship between smoking and mood; goal setting; identification of triggers to smoke; and facilitating problem solving and skills building, including the use of mood management strategies that also act to aid cessation (e.g., exercise, scheduling pleasant activities). A dedicated Quitline counselor will manage the quitting process for each participant.
- As in the control condition, with consent, a letter will be sent to the person's GP and/or psychiatrist. Additionally for the intervention condition, peer reviewed articles that provide practical advice to assist doctors in helping people with mental illness to quit smoking will be included (36, 37). Additionally, participants will receive a Quit Victoria brochure for carers and a Quitting Mood and Experiences Diary.
- Quitline engagement with mental health services: Quitline will provide written feedback to treatment providers at the end of the telephone counseling program. Providers will be encouraged to monitor and support cessation efforts whenever appropriate. In addition, Quitline will contact the mental health treatment provider, if concerns arise about mental health issues.
- NRT: Participants will initially be provided with a 4 week supply of patches (one 21 mg patch/day) plus their choice of an oral-form NRT (gum, lozenge, inhalator, spray). The research team will post NRT to participants with an information pack that includes printed instructions on how to use NRT correctly, for how long, potential side effects (and when to notify a health care provider), and safe storage and handling. Quitline counselors will monitor and encourage correct use of NRT and address barriers to use. Intervention participants that decide to use the supplied NRT will receive a final 4-week supply of NRT as per the initial supply. Quitline counselors will ask participant preferences for oral dose forms during the Week 2 call (for those participants that do not engage with Quitline, the peer worker will attempt to contact participants to determine participant preferences for NRT) in order for NRT to be delivered to the participant by Week 4. Participants who desire to shift to use of a prescription-based stop smoking medication (e.g., varenicline) will be supported to do so, but the study will not fund the purchase (which is low for those with health care cards as it is heavily government subsidized).

The Quitlink intervention is similar to the Quitline's routine care for clients disclosing mental health issues. Components unique to this trial include the peer worker referring to Quitline, a dedicated Quitline counselor for each participant and provision of NRT.

### Discontinuation of the Quitlink Intervention

This may occur if there are alterations in the participant's condition which justifies the discontinuation of treatment in the investigator's opinion. All intervention components are voluntary and non-essential to participation. The participant may refuse to engage, miss scheduled telephone sessions, or discontinue with the Quitlink intervention without affecting study participation.

NRT use is also optional (recommended, but not expected), and use or non-use will not affect whether they can participate in Quitline counseling or follow-up interviews.

### Intervention Training and Supervision

Quitline counseling delivery will be provided by existing Quitline Victoria counselors, holding at least a Certificate III level qualification in counseling and trained in the World Health Organization Smoking Cessation approach (38) by a Quit psychologist. In addition, all are experienced in conducting smoking interventions among SSMI and in 2014 received a 1-day face to face training workshop led by experienced investigators focusing on the provision of structured monitoring of mood, nicotine withdrawal, and psychiatric medication side-effects. It is standard practice for the Quitline counselors to receive monthly group supervision led by a qualified counselor and monthly individual supervision which entails a psychologist reviewing notes and listening to two calls (<10 and >20 min) to facilitate reflective practice and quality assurance. Counselors will also receive a minimum of 1 day of additional training focused on refreshing these skills and processes contained in the Quitlink treatment manual. The Quitlink treatment manual developed for this study will be used to ensure that all participants receive a minimum standard of behavioral counseling, are supported to use NRT therapy provided and that communication with mental health services occurs as necessary.

Peer workers will identify as being or having been a mental health consumer and preferably will also be ex-smokers or non-smokers who have experience working with SSMI and are aware of the challenges involved in smoking cessation for this group. Peer workers will receive training and ongoing supervision from investigators experienced in working with peer workers (including investigators with lived experience). Training and supervision will cover recruitment issues, delivery of standard care (brief intervention), baseline assessment, the automated randomization procedure, how to refer to Quitline, fidelity, distress management procedures, and suicide risk assessment and referral.

### Intervention Fidelity

For purposes of the present study, a random selection of 20% of Quitlink intervention participants will be made, and routinely recorded calls will be rated for fidelity to the treatment manual by an independent rater using a checklist derived from the treatment manual which includes core behavior change techniques (BCTs) relevant to smoking cessation. BCTs are defined as the smallest identifiable components of an intervention that in themselves have the potential to change behavior (39).

### Concurrent Treatment

In both the Standard Smoking Care and Quitlink Intervention conditions, participants will be able to partake of any interventions initiated by themselves or their health providers during the course of the study and these will be monitored at the follow-up assessments.

### Outcome Measures

Outcome measures will be assessed at 2-, 5-, and 8-months post baseline, by telephone. These will be conducted by independent assessors who will remain blind to intervention allocation. Outcome measures will all be assessed before any process measures where answers could suggest experimental condition. All assessment instruments are widely used in mental health and/or tobacco treatment research and practice (see Table 1) and cover the domains hypothesized to be impacted upon by the intervention.

**Table 1:** Assessment schedule.

**Assessments**	**Baseline**	**2 month**	**5 month**	**8 month**
***Demographic characteristics***	X			
**MENTAL ILLNESS DIAGNOSIS**				
Self-report -Have you ever received a mental health diagnosis? -Have you ever been diagnosed with a psychotic disorder?	X			
MINI (diagnostic interview)		X	^*^	^*^
McLean screening instrument for borderline personality disorder		X	^*^	^*^
**MEDICATIONS**				
Current medications	X/E			
Medication side effects	X			
**SMOKING MEASURES:**				
Current smoking and quit attempts	X	X	X	X
7 day point prevalence abstinence (self-reported)		X	X	X
6 month prolonged abstinence (primary outcome)				X
CO Monitoring (those reporting abstinence)				A
Heaviness of Smoking Index	X	S	S	S
Tobacco types	X			
Cost	X	S	S	S
History (age first smoked)	X			
Social influences	X			
Cravings	X	X	X	X
Smoking use motives	X			
Situations not allowed to smoke		X	X	X
Goal	X			
Motivation to quit	X	S	S	S
Confidence to quit	X			
Self-efficacy		X	X	X
Products/services to help quit (including NRT, Quitline)	X	X	X	X
Nicotine replacement products (helpfulness, likely use)	X			
Counseling preference (in person or telephone)	X			
Minnesota Nicotine Withdrawal Scale (only two items in follow ups)	X	X	X	X
**MENTAL HEALTH:**				
Kessler-10	X	X	X	X
**SUBSTANCE USE:**				
Alcohol (AUDIT-C)	X	X	X	X
Cannabis use with tobacco question	X	X	X	X
Cannabis (First question of CUDIT-R)	X	X	X	X
**QUALITY OF LIFE:**				
AQoL-8D	X	X	X	X
**MEDICATIONS—NRT/CESSATION:**				
Process measure (i.e., provided to intervention participants)		E		
***Perceived support—***GP, Psychiatrist, other health professional		X		
**QUITLINE USE:**				
Number, length, content and timing of calls		E		
**SERVICE USE**				
Hospitalizations and other intensive health service use		X		X
Time off from work and usual duties		X	X	X
Financial stress questions	X	X	X	X
**Therapeutic Alliance:**		X		
WAIT-3		X^$^		
Peer worker brief intervention question		X		
**Qualitative interviews**		I	I	I
**PBS/MBS cost data**				E

*AUDIT-C, Alcohol Use Disorders Identification Test – Brief (40); AQoL-8D, Assessment of Quality of Life-8D (41); CUDIT-R, Cannabis Use Disorders Identification Test – Revised (42); CO, Carbon monoxide; GP, General Practitioner; Kessler-10, Kessler Psychological Distress Scale (43); MINI, Mini International Neuropsychiatric Interview (44); NRT, Nicotine Replacement Therapy; WAIT-3, Working Alliance Inventory for Tobacco-−3 (45); MBS, Medicare Benefits Schedule; PBS, Pharmaceutical Benefits Scheme*.

**Key**

* *If not captured at previous assessment*.

***E** Extracted data*.

***S** Current smokers*.

***$** If used Quitline*.

***A** Those reporting abstinence*.

***I** Selected subsample*.

#### Primary Outcome

The primary outcome is defined as continued abstinence from smoking since the end of the treatment period, i.e., 6 months sustained abstinence, with no relapse (defined as 7+ days of continuous smoking, and no reported smoking in the last week), with biochemical verification at 8-month follow-up. Sustained abstinence will be assessed via the following question: “When did you last smoke a cigarette, even a puff?” If a participant reports prolonged abstinence at the 8 month follow up, and no smoking in the last week, they will be asked to attend a face to face visit to complete CO testing for objective validation using a Micro+ Smokelyzer, with a reading of 8 ppm or higher defined as indicative of recent smoking.

#### Secondary Outcomes

##### Smoking

Secondary outcomes assessed at 2-, 5-, and 8-months post baseline will include:

7-day point prevalence abstinence, based on “Have you smoked at least part of a cigarette in the last 7 days?”
Reported cigarettes smoked per day (for daily smokers) or cigarettes per week (for non-daily smokers).
Expenditure on cigarettes.
Number of quit attempts of 24 h or more, 1 week or more, and 1 month or more in the previous 3 months or since last assessed.
Time to relapse: in those who do relapse will be determined by asking when they first smoked after a quit attempt.
Number of subsequent quit attempts among those who relapsed.
Hospitalizations and other intensive health service use.
Financial stress questions adapted from Siahpush and Carlin (46).
Productivity impacts (time off work or other duties).

Heaviness of Smoking Index (HSI): Nicotine dependence is assessed using this two item Index (47, 48). It uses a six-point scale calculated from the number of cigarettes smoked per day (1–10, 11–20, 21–30, 31+) and the time to first cigarette after waking (≤5, 6–30, 31–60, and 61+ min). Nicotine dependence is then categorized into a three-category variable: low (0–1), medium (2–4), and high (5–6). The HSI has been found to have good reliability and reasonable predictive validity (49).

Cravings: assessed by one item taken from the International Tobacco Control (ITC) Four Country Survey (50, 51), “Currently, how often do you get strong cravings to smoke tobacco?” with the response options of: (1) Hourly or more often; (2) Several times per day; (3) At least once a day; and (4) Less than daily. Difficulty in coping with situations in which smoking is not allowed is also assessed, on a 4-point Likert Scale from “very,” “moderately,” “mildly” to “not at all difficult.”

Withdrawal symptoms: as assessed by the Minnesota Nicotine Withdrawal Scale [MNWS; (52)], an eight item ordinal scale rating withdrawal symptoms from 0 (not present) to 3 (severe). At baseline the MNWS is administered, with two symptoms (concentration and appetite) assessed at follow-up. The MNWS has been shown to have good reliability and predictive validity (53).

##### Mental health

Kessler Psychological Distress Scale [Kessler-10; (43)] a 10-item scale of non-specific psychological distress. Low scores (10–15) indicate little or no psychological distress and higher scores indicate increasing levels of distress (moderate, 16–21; high, 22–29; and very high, 30–50). It has shown consistent psychometric properties across major sociodemographic subsamples (54).

##### Substance use

The Alcohol Use Disorders Identification Test—Brief [AUDIT-C; (40)] a three item screening tool used to identify hazardous alcohol use or active alcohol use disorders. It is scored on a scale of 0–12 with a cut off of 3 (women) or 4 (men). For men, it has been shown to have a sensitivity of 0.90 and specificity of 0.45; for women the sensitivity is 0.80, and specificity is 0.87.

The Cannabis Use Disorders Identification Test—Revised [CUDIT-R; (42)] is a briefer (8-item) and more refined version of the CUDIT (55), a simple modification of the AUDIT. Items cover the domains of consumption, cannabis problems, dependence, and psychological features. The CUDIT-R was found to comprise a single factor, with high test-retest reliability (*r* = 0.871), high internal consistency (α = 0.914), and discriminant validity (area under the curve = 0.960). Only question 1, “How often do you use cannabis? (over the last 2 months)” is included in the present study (never, monthly or less, 2 to 4 times a month, 2 to 3 times a week, 4 or more times a week). In addition, participants who use cannabis are asked “Do you ever mix tobacco with your cannabis?” with response options of “Yes, always or nearly always,” “Yes, sometimes” or “No, never or very rarely.”

##### Quality of life

The Assessment of Quality of Life 8 Dimension [AQoL-8D; (41)] instrument is comprised of 35 items from which eight dimensions (independent living, pain, senses, mental health, happiness, coping, relationships, and self-worth) and two “super-dimensions” (physical and psychosocial) are derived. It has demonstrated strong content validity and has been found to perform relatively well in populations with SMI (56). The 35 items may be reduced to a single utility score. Use of the instrument enables calculation of quality adjusted life years (QALYs) experienced across the two study arms, which will be reported in the cost-effectiveness analysis.

##### Covariate or process measures

Demographic variables (e.g., gender and age).

History of tobacco smoking and quitting.

Types of tobacco used.

Social influences on smoking, e.g., lives with other smokers.

Smoking Use Motives: As part of this trial self-reported reasons for smoking are assessed using a modified version of the Drinking Motives Questionnaire (57), with additional items developed by Spencer et al. (58) to explore the use of substances to alleviate psychotic symptoms (positive and negative).

At baseline, participants will be asked whether they have a preference for in person or telephone counseling. They will also be asked to rate the likely helpfulness of NRT to long term quitting (not at all, some, moderately, extremely) and likelihood of use in the longer term (not at all, some, moderately, extremely).

Motivation to quit: assessed by a single question adapted from Crittenden et al. (59), “How much do you want to quit smoking?” (not at all, a little, some, very much). At follow-up assessments, “Are you trying to quit smoking altogether or are you planning to keep smoking at this level?”

Confidence to quit (at baseline) is measured by the following question: “How confident are you that you can stop smoking for good in the next 2 months if you wanted to?” (not at all, somewhat, moderately, very, extremely).

Self-efficacy in quitting is measured by the following question adapted from Perkins et al. (60): “How confident are you that you will not smoke at all tomorrow?” (not at all, somewhat, moderately, very, extremely). For those who quit at follow-up, “How confident are you that you will be able to stay quit long-term and become a permanent ex-smoker?” (not at all, somewhat, moderately, very, extremely).

Medications: Changes in use of prescribed psychotropicmedication.

Medication side effects: At baseline, participants will be asked to rate 10 symptoms during the past week (e.g., dry mouth, increased thirst) on an ordinal scale from 0 (not present) to 3 (severe). This measure is informed by the most common adverse side effects of psychiatric medications as identified in the Side Effect Survey, which has demonstrated validity and reliability (61).

Treatment received (use of NRT and Quitline—number and length of calls).

Objective data on service use (number and length of calls) will be extracted from the Quitline database for all participants (as some control participants may have self-referred).

Therapeutic alliance with Quitline counselor: the three-item Working Alliance Inventory for Tobacco (45), measuring goal, task, and bond on a five item Likert Scale (seldom, sometimes, fairly often, very often, always) will be administered at the 2-month follow-up. The three-item measure has been found to have acceptable-good internal consistency and construct validity.

Self-reported service use and satisfaction: participants' use and assessment of level of support they have received for quitting from their mental health service, doctors, and other health professionals.

Linked data on service and prescription medication use from the Australian Government subsidized Medicare and Pharmaceutical Benefits Schemes.

##### Safety data

Adverse events will be collected at all follow-up time points, with prompting via questions asking how the participant has been feeling in general and if they have any health concerns.

### Sample Size Determination

Based on our previous study (23) which achieved a 15% prolonged abstinence rate in depressed smokers, and knowing rates are considerably less among those with more severe mental illnesses (62) we anticipate that for the primary outcome of prolonged abstinence at 8 months, prolonged abstinence will occur in 1% of the control arm vs. 8% in the intervention arm. To detect this effect with 80% power at *p* = 0.05, we require 134 per arm. We expect ~30% attrition, inflating the sample size to 191/arm or 382 overall. Thus, we will recruit 382 smokers over 36 months and follow up at 2-, 5-, and 8-months post-baseline, completing the study over a 4.5-year period.

### Participant Recruitment and Retention

Peer workers will visit sites and provide information to both staff and potential participants about the study to encourage recruitment. Service staff will be asked to refer potential participants (at any stage of their treatment) to the study via the peer worker. Additional recruitment strategies will be by advertising (e.g., flyers, newsletters, online via service websites) and peer workers attending community meetings and other events to inform potential participants directly about the study to encourage self-referral.

For those who meet eligibility criteria and decide to participate in the study, the peer worker will gain written informed consent from the participant. Provision is made on the consent form for opting in or out of possible participation in a qualitative study of experiences of trying to stop smoking and for participation in further studies.

Telephone follow-up and compensation for completed assessments will aid in increasing retention rates. Monthly check-in texts (to remind participants to inform the researchers if their contact details change) will be conducted to help maintain contact with participants, and 3 monthly follow-up will assist with accurate participant recall of smoking and quitting history. Participants will receive a $40 gift card for baseline and for each completed follow-up assessment and a $40 gift card for the 8-month face-to-face assessment for biochemical verification of self-reported smoking cessation (if required).

Upon completion of the baseline assessment, the peer worker will provide standard smoking care (described above) to all participants. The peer worker will then access the randomization allocation for the participant via the eCRF program, and communicate appropriately with the participant.

### Randomization

Following completion of the baseline assessment, a brief intervention will be delivered by a peer worker—prior to randomization—to ensure all participants receive the recommended minimum standard care, in a manner that is unbiased by the outcome of randomization. Following this, the computer program used to complete the baseline will randomize to condition using 1:1 randomization. Participants will be randomly allocated to either no further intervention, or to be contacted by Quitline who will offer a targeted callback counseling intervention with NRT provided, over an 8-week period. As stated above, cluster randomization will be used in situations where risk of contamination is higher, such as residential services, stratified by short- or long-term residence, with 1:1 allocation. Individual randomization will be used in services where contamination of risk is lower, via permutated block sizes of 4 and 6 to avoid incomplete blocks, stratified for site. Participants will be allocated a unique computer-generated study number. Randomization will be independently managed by the trial epidemiologist (JA) and uploaded to a web-based data capture tool (Research Electronic Data Capture; REDCap) that will also have case report forms (eCRF) created for the project using REDCap.

Following randomization, those in the intervention group will be told of the additional supports they will be getting (see above). Controls will be simply told the session is over and reminded of when the first follow-up survey might be expected.

### Blinding

Outcome assessors will be blinded to study design and allocation and will have training and regular supervision on practices to maintain blinding in a PROBE design study. These have been previously used successfully by our team (63). Importantly, outcomes assessors will ask participants about smoking outcomes prior to any questions about use of cessation supports, questions that often produce answers which can indicate likely experimental condition.

The mental health practitioners, follow-up assessors, qualitative interviewers, and Quitline counselors are located in separate organizations, which will maximize maintenance of the outcomes assessors' blindness to study design and treatment allocation. Outcomes assessors will access only contact details, and not treatment files. The eCRF permissions will not allow outcomes assessors to access information about the participant's treatment allocation.

Participants will be aware of what support they are receiving, but not of the comparison condition due to the “limited disclosure” approach. Participants will be informed about what is involved (i.e., the follow-up assessments) and that they may be offered support with smoking cessation. They will not be informed of the specifics of the support (i.e., intervention will receive proactive referral to Quitline and be supplied with NRT). Control participants will be informed of the options available and encouraged to follow up on any they are interested in, in the usual manner (GP/other health professional/self-referral to Quitline). The outcomes assessment team will remain blinded to treatment allocation until completion of the study. Data analysts will be blinded by labeling the intervention conditions “A” and “B.”

### Unblinding

Following baseline assessment and delivery of the brief intervention, peer workers, the trial coordinator, quitline counselors, qualitative staff, and associated investigators will be unblinded to treatment allocation**.**

### Stepwise Procedures

This protocol is presented in accordance with the 2013 SPIRIT (Standard Protocol Items: Recommendations for Interventional Trials) Statement (see Supplementary Material). The schedule of enrolment, interventions, and assessments is summarized in Table 2.

**Table 2:** Stepwise procedures.

**Contact/visit**			**Intervention period**								**Follow-up period**							
**Week**	**−1**	**0**	**1**	**2**	**3**	**4**	**5**	**6**	**7**	**8**	**9**	**10**	**…**	**21**	**22**	**…**	**34**	**35**
Visit number		1									2			3			4	
**ENROLLMENT**																		
Screening (inclusion/exclusion)	X	X																
Informed consent	X	X																
BASELINE ASSESSMENT		X																
Standard smoking care		X																
Randomization		X																
INTERVENTION^*^																		
Referral to Quitline		X																
Contacted by Quitline and smoking cessation initiated			X															
Quitline determines preferred oral NRT for second 4-week supply				X														
NRT dispensing		X		X														
Smoking cessation program			X	X	X	X	X	X	X	X								
FOLLOW-UP ASSESSMENTS											X			X			X	
Blinded follow up assessment conducted (all participants)											X			X			X	
Potential qualitative interviewees identified^**^											X			X			X	
Qualitative interviews conducted^**^												X			X			X
CO monitoring (on reported abstainers)																	X	
**ADVERSE EVENTS**																		
Unprompted (serious/severe)			X	X	X	X	X	X	X	X								
Prompted (all)											X			X			X	

* *Intervention group only*.

** *Participants will be purposely selected at each of the assessment timepoints (2, 5, and 8 months) to be invited for interview*.

### Data Management

All data will be entered electronically via eCRF using (REDCap) tools (64) hosted at Hunter New England Local Health District on a secure server. Redcap is a secure, web-based application designed to support data capture for research studies providing an intuitive interface for data entry, audit trails for tracking data manipulation and export, automated export procedures for downloads to statistical packages and procedures for importing data from external sources. The lead investigator (and/or delegate) and study coordinators will conduct ongoing data checking and cleaning.

Participants' personal details will be accessed, used, and stored according to relevant legislations. Access to external health data (e.g., Quitline, MBS/PBS, health records) will only occur with the consent of the participant in accordance with protocols of relevant external agencies (e.g., Commonwealth Department of Human Services for MBS/PBS data). The trial conduct and safety data will be monitored by a Data Safety Monitoring Board (DSMB).

### Statistical Methods

#### Primary and Secondary Outcomes

Independent and blinded statisticians from the CReDITSS Unit at the Hunter Medical Research Institute, Australia, supervised by AI Attia, will conduct analyses of the primary and secondary outcomes.

Analyses will be carried out using a cluster randomized trial framework where the individuals (*n* = 150) are treated as clusters that contribute only one person, the short-term residential programs are clusters that contribute an average of 15 people each (10 programs × 15 people/program = 150 total) and the long-term programs are clusters that contribute 10 people each (6 programs × 10 people/program = 60). We will use a generalized linear mixed model (linear regression for continuous outcomes and logistic regression for dichotomous outcomes) to handle the clustering and the repeated measures at baseline, 2-, 5-, and 8-months; individuals will be modeled as random effects, cluster as a random effect, and group assignment as a fixed effect. Mixed models allow for missing data for the primary intention to treat analysis, but a sensitivity analysis using a worst case scenario (baseline value for continuous outcomes or relapse for dichotomous outcomes in case of missing value) will also be carried out.

Intervention participants who do not complete the intervention, and participants who miss an assessment follow-up time point, will be kept in the study and contacted for later assessments (unless they choose to withdraw from the follow-up assessments).

#### Exploratory Analyses

We plan to examine whether the amount of intervention (Quitline counseling, NRT) received by participants is related to outcomes. We will also explore different imputation strategies for missing data related to outcomes.

#### Economic Evaluation

A cost-effectiveness analysis of Quitlink will be conducted alongside the trial described here, using data 8 months post randomization. A modeled analysis will estimate future costs and benefits of smoking cessation beyond the trial period, over the life course. Full protocol details of this are presented in this Special Issue in Sweeney et al. (in submission). In brief, incremental cost-effectiveness ratios (ICER) will be calculated for the cost ($AUD) per successful quit and quality adjusted life year (QALY) gained (i.e., cost-utility) as a result of Quitlink when compared with usual care. Healthcare system and limited societal perspectives will be taken.

### Qualitative Evaluation

A nested qualitative study will be conducted. All interviews (participants and workers) will be audio recorded, transcribed, and a general inductive approach will be taken to the analysis (65).

#### Participant Interviews

Semi-structured individual in-depth interviews will be conducted with 72 participants. The (unblinded) qualitative researchers will access assessment data (REDCap) to view participants' cigarette consumption and service use data. They will use these data to purposively invite participants at each of the assessment timepoints (2-, 5-, and 8-months) for interview. Potential participants will be sent a flyer via text, mail, or email (depending on contact information available) asking them to participate in an interview. Upon affirmation, they will be provided the full Information Statement on the interview component.

#### Participant Selection

At 2 months, 30 participants will be interviewed (15 in each study arm, with cessation outcomes balanced across groups to negate any potential therapeutic effect of the additional qualitative interviews). The majority of the interviews (~10 per arm) will be with participants who have either not reduced smoking levels or have made only some reduction in smoking (<50%). Having a mix of those who have engaged with the intervention (attended 4+ Quitline sessions; or engaged in other treatments) and those who have not engaged or under-engaged (1–3 sessions) will enable identification of both barriers to engagement and barriers to change.

At 5 months, another 30 participants will be interviewed (15 per treatment arm, balanced for smoking outcomes). The majority (~10 per arm) will be “relapsers” (defined as those who have reduced consumption by 50–100% at 2 months but have resumed or increased use by 5 months), to allow a focus on medium term barriers to cessation maintenance, again with a mix of those who are engaged and those who are non- or under-engaged.

At 8 months, 12 participants will be interviewed (no balancing required as this is after primary endpoint collection), including ~6 who have relapsed (in order to focus on longer term barriers to cessation maintenance). At each time point, interviews will also be conducted with people who have successfully quit and maintained cessation to determine whether those who are successful face the same barriers as others but overcome them, and/or use the intervention in different ways. Participants in the qualitative study will be remunerated $40 for participation in individual interviews expected to take ~45 min.

#### Mental Health and Quitline Victoria Counselor Interviews

Interviews will evaluate the acceptability of the intervention among mental health practitioners (including peer workers) and Quitline counselors. Semi-structured individual in-depth interviews will be conducted with 15 mental health practitioners to enable data collection to reach saturation and for key themes to be identified (66). For Quitline counselors, three group interviews (4–5 counselors per group) will be conducted. Interviews with mental health practitioners and Quitline counselors will explore their experience of the program and its strengths and weaknesses from their perspective. Mental health practitioners will also be asked to focus on the implementation and sustainability of the Quitlink intervention.

### Data Safety Monitoring Board

A Data Safety Monitoring Board (DSMB) will be established. The DSMB will monitor safety and adverse events reported and will convene as required throughout the duration of the trial. The DSMB will be composed of individuals with appropriate expertise (e.g., clinical trials, statistical expertise, mental health expertise) who are independent from the study and free of conflict of interest, and where this is not practical, measures will be taken to minimize the perceived conflict of interest. The DSMB will have the capacity to contribute to decisions regarding continuation or discontinuation of the trial based on scientific and ethical factors. The DSMB will operate under the rules of an approved charter that will be written and reviewed by the DSMB. Each data element that the DSMB needs to assess will be clearly defined in the DSMB charter. The DSMB will provide its input to the Chief Investigator, and this will be reported to HRECs and other regulatory bodies as per local guidelines.

### Safety Monitoring

Adverse events (AEs) will be collected and reported as per Good Clinical Practice guidelines, from the point of enrolment until end of their participation. Follow-up assessors will prompt for all AEs as part of the interview schedule, while Quitline counselors will record any serious or severe events (SAEs) reported during counseling, as per current Quitline protocols, and will report these to research staff. If a participant withdraws from the study with an ongoing AE during the treatment phase, AEs will be followed up until it is resolved; or 7 days following withdrawal, at which time participants will be advised to contact their treating physician if AEs persist. The DSMB will review safety data on a regular basis, with SAEs and other significant safety issues reported immediately to the DSMB and further (e.g., governing ethics committee/s) as necessary as per local guidelines.

## Anticipated Results

This RCT will test the effectiveness of the Quitlink intervention for smoking cessation among SSMI. We anticipate that the intervention will be associated with significantly higher rates of continued abstinence from smoking at 8-month follow-up, relative to the control condition. We also anticipate the intervention will be more cost-effective compared to the control condition of usual care and reduced financial stress for participants. Using qualitative methods, we will also identify barriers and enablers to making and sustaining quit attempts. A range of secondary outcomes will be measured on follow-up occasions and we expect that the Quitlink intervention will be associated with significantly better outcomes on these variables (higher rates of 7-day point prevalence abstinence, quit attempts, smoking cessation motivation and self-efficacy, mental health, and quality of life and lower reported cigarette consumption, nicotine withdrawal symptoms, expenditure on cigarettes, and alcohol, and cannabis use).

## Ethics and Dissemination

Prior to participation in the trial, the person will be fully informed about the research and given ample time and opportunity to enquire about details and decide whether or not to participate. If they agree to participate they will be asked to sign the study specific consent form. To ensure anonymity and to limit disclosure, participants will be assigned a unique identifier at the time of randomization. Results arising from the RCT will be published in peer-reviewed journals and disseminated at international conferences. Results will be reported in such a way that participants will not be identifiable.

### Research Ethics Approval

Ethics approval has been obtained through St Vincent's Hospital, Melbourne (HREC Reference Number: HREC/18/SVHM/154), the University of Newcastle HREC (HREC Reference Number: H-2018-0192) and the Cancer Council Victoria, HREC (HREC Reference Number: 1807).

### Protocol Amendments

Each study site will only be able to start data collection once the relevant Ethics Committee approval is obtained. In the case of proposed protocol changes, an amendment will be submitted to the Ethics Committees for approval, and the trial coordinating center will ensure all study staff are provided with new documentation. Any significant protocol changes will be updated on the ANZCTR and reported in the final outcomes paper.

### Consent or Assent

The study is based on the principles of Good Clinical Practice according to the Declaration of Helsinki. Potential participants will be given oral and written explanation of the study including the potential risks, their right to withdraw at any time and the details of data protection and confidentiality and sufficient time to ask questions. A signed consent form will be obtained. Participants will be given the opportunity to agree or decline to being contacted for ancillary studies, without effecting participation in the main trial. A copy of the PICF will be given to the person.

### Confidentiality

The trial will be conducted in accordance with applicable Privacy Acts and Regulations. All information regarding trial participants will be treated in strict confidence. Participants' identifying details will be stored separately from other data. Participants will be informed of the potential reasons for breaching confidentiality in the PICF (risk of harm to self or others). Data, which identify any trial participant, will not be revealed to anyone not directly involved in the trial or the clinical care of that participant.

### Access to Data

All data will be considered the property of the trial chairperson, who, in consultation with the trial management committee, will be responsible for presentations and publications arising from this trial.

### Dissemination Policy

Trial findings will be summarized and posted to participants who have indicated they would like a copy of the results.

## Discussion

The Quitlink study is the first rigorously designed RCT to evaluate a specialized quitline intervention accompanied by NRT, for people with SMI, with biochemical verification of self-reported abstinence. Accessible smoking interventions like quitlines are clearly required to improve the mental and physical health of smokers in receipt of mental health treatment and links with mental health services are crucial to ensure maximum utilization. A major strength of this study is that it is demonstrably an intervention that can and will be used if the trial demonstrates it helps: it uses two strategies that are currently funded, i.e., quitline and peer workers, but not currently co-ordinated. Quitlines exist but are underutilized by those with SMI; likewise, peer workers are employed but do not uniformly see smoking cessation as part of their role. The study investigates a model for how these two existing strategies can be co-ordinated to maximize the health impact for SSMI, who often wish to quit but are not properly supported to do so. Having peer workers trained in assessment, brief smoking cessation advice, and proactive referral to quitline is more likely to attract SSMI to consider smoking cessation. It is a simple and potentially cost effective method of increasing access to smoking cessation services in the mental health sector.

### Limitations

There are three main limitations associated with this trial. Firstly, due to cluster randomization of residential sites, the peer workers will become aware of each site's allocation. Peer workers will be carefully trained and supervised not to communicate this information. They will also be supervised so as to encourage equal recruitment across control and intervention sites. Secondly, outcome to 8-months has been chosen as the focus of this study so as to examine medium term smoking, which parallels that for well populations (67). However, it would be informative to follow the sample over a longer timeframe to measure longer-term health and other benefits.

### Conclusions

If Quitlink is shown to be effective, it has the potential to greatly improve individuals' longevity, quality of life, mental health, and reduce health care costs. This is an innovative and practical service delivery model that has the potential to ensure that smokers with SMI have access to best-practice smoking cessation treatment. Secondly, regardless of effectiveness outcomes, the project's qualitative study will provide greater insights into the barriers faced by smokers with SMI and will assist in the development of even more effective interventions.

The intervention can be quickly and directly translated to quitlines and mental health services, to improve rates of smoking cessation among SSMI. Study findings will be of significant interest to consumer and carer groups, the broader community sector, as well as researchers and clinicians. The rigorous study design, inclusion of cost-effectiveness evaluation and qualitative study are key strengths.

## Author Contributions

The first draft of the paper was written by AB with significant input from KM and AT before receiving input from remaining authors. The study was conceived and designed by all authors.

### Conflict of Interest Statement

The authors declare that the research was conducted in the absence of any commercial or financial relationships that could be construed as a potential conflict of interest.
